# A dataset of harmonized global air quality monitoring metadata

**DOI:** 10.1038/s41597-026-06797-0

**Published:** 2026-02-17

**Authors:** Stefania Renna, Carlos Rodriguez-Pardo, Lara Aleluia Reis

**Affiliations:** 1 Polytechnic University of Milan, Department of Management, Economics and Industrial Engineering, Milan, 20156 Italy; 2 CMCC Foundation - Euro-Mediterranean Center on Climate Change, Lecce, 73100 Italy; 3 RFF-CMCC European Institute on Economics and the Environment, Milan, 20144 Italy; 4 NOVA School of Science and Technology, NOVA University Lisbon, Campus de Caparica, 2829-516, Caparica, Portugal; 5 CENSE – Center for Environmental and Sustainability Research & CHANGE - Global Change and Sustainability Institute, NOVA School of Science and Technology, Universidade Nova de Lisboa, Campus de Caparica, 2829-516, Caparica, Portugal

**Keywords:** Environmental monitoring, Environmental impact, Pollution remediation

## Abstract

This study addresses the gap in air quality monitoring metadata reporting by building a classifier for air quality station types and area characteristics. It leverages ultra-high-resolution land cover data, complemented by additional demographic and gridded information. We employ advanced machine learning methods, including convolutional neural networks and transformers. Through a custom training approach, we fine-tune pre-trained models on 7000 images and label +8000 additional monitors, resulting in a robust model for classifying air quality stations by area characteristics (urban, rural) and source type (background, non-background). The result is a global harmonized dataset of governmental air quality station metadata for particulate matter, with  ~ 15000 monitors from 106 countries. For each station, the dataset provides an identifier, geographical coordinates, country, area characteristics, source type, and classification status. This dataset enables global feasibility studies and regional analyses of conditions leading to exposure. By providing a consistent classification of monitoring stations, it also allows for meaningful comparisons of sectoral exposure contributions across countries, regions, and station types, supporting comparative studies and health impact assessments.

## Background & summary

Air pollution, especially through fine particulate matter (PM_2.5_), is a threat to human health globally, contributing to millions of premature deaths every year^1–3^. Governments’ environmental agencies and local authorities deploy ground-level monitoring networks to assess and manage ambient air quality aiming to lower population exposure to hazardous levels of air pollution. Air quality monitoring networks often include metadata describing the characteristics of each station, such as its location, elevation, degree of urbanization, and dominant pollution sources. These are essential for interpreting exposure patterns. Stations are typically classified according to two main dimensions: area characteristics (e.g., urban, sometimes suburban, or rural) and source type (e.g., background, traffic, or industrial). This classification is relevant for ensuring meaningful comparisons across different locations and over time. It helps distinguish between pollution from local sources, such as traffic or industry, and regional background pollution, among others. For example, background air pollution levels, representing the level of pollution not directly attributable to local sources such as traffic^4^, play an essential role in defining baseline population exposure. This classification is especially relevant when assessing the contribution of different sectors to air quality^5^, designing mitigation policies, and conducting air pollution exposure and health impact assessments. However, there is currently no consistent global standard for this classification, limiting comparability across countries and regions. Moreover, despite rapid expansion in some emerging economies, many regions still lack adequate monitoring coverage and structured metadata^6,7^, particularly in middle- and low-income countries^8^.

The absence of this type of information limits the ability to conduct comparable air pollution exposure assessments and to analyze exceedances, particularly in relation to the updated World Health Organization’s (WHO) Global Air Quality Guidelines for PM_2.5_^1^. Although alternative data sources such as radar and satellite observations are now available, ground-based monitoring stations are widely regarded as the gold standard in air quality assessment because they undergo regular maintenance and calibration, ensuring high accuracy and reliability of the data they provide^9^.

To take advantage of ground-level air quality measurements, we develop a method to classify available monitoring stations worldwide. We apply advanced machine learning techniques, including convolutional neural networks (CNNs) and vision transformers, to perform this classification. Despite recent progress in computer vision, this task remains challenging because the criteria for siting monitoring stations vary significantly between countries^10^. For example, in China, national-level monitors are often placed in cleaner areas, while local monitors tend to be located in more polluted zones, reflecting different policy priorities and monitoring strategies. While distinguishing between traffic-related and industrial sites can be relatively straightforward for deep learning models, separating urban background stations from those influenced by traffic or industrial activities is far more challenging. In urban areas, pollution sources are diverse and often overlap, making it difficult to clearly differentiate background from non-background stations.

Few studies exist in the literature on the classification of air quality stations. For instance, some studies focus on predicting the optimal number of stations in a network^11,12^, some apply categorization based on historical data^13,14^, while others focus on past pollution and meteorology^15^, or classify and predict air pollution based on collected real-time environmental data^16^. To our knowledge, no previous work applies deep learning methods to predict air quality station metadata information at the global level.

This study addresses the gap in air quality monitoring metadata accounting by building a classifier of air quality station area characteristics and types. It leverages the European Space Agency (ESA) WORLDCOVER’s 10m resolution land cover data, complemented by additional information, such as air pollution, population, and industrial plant information. We employ state-of-the-science computer vision models including CNNs and Vision Transformers. With a custom training methodology, we fine-tune pre-trained models on about 7,000 images while labeling more than 8,000 additional monitors, establishing a robust model for classifying air quality stations by area characteristics (urban, rural) and source type (background, non-background). This enables the creation of Metair, the first global harmonized dataset of air quality station metadata for particulate matter, with about 15,000 monitors from 106 countries. For each air quality station, the dataset provides an identifier, geographical coordinates, the associated country, source type, area characteristics, and classification status (official vs. estimated). Our classification system is grounded in the European Environment Agency’s (EEA) labeling framework^17^, as it is well-defined and provides enough data to effectively train our models.

This work offers both methodological and practical contributions. This dataset will serve for global-level feasibility studies and regional characterizations of environmental conditions leading to chronic background exposure, as well as comparative studies across different areas and source types, or for estimating health impacts. We introduce a deep learning algorithm and architecture that leverages fine-tuning of pre-trained image classifiers and a self-attention-based data fusion mechanism that enables our model to account for visual information and metadata simultaneously. We provide a scalable and extendable tool that not only facilitates the classification of unclassified and newly introduced governmental air quality stations, but holds potential applications for labeling low-cost sensors complementing institutional air quality networks^18,19^ as well as those installed in citizen science campaigns.

## Methods

In this Section, we introduce our model design and training methodology. In particular, we describe the input data fed into the models and present a novel approach to predict air quality stations’ metadata. In the Data Records Section, we present our dataset. In the Technical Validation Section, we discuss model experiments and limitations of our work.

### Data collection and sources

#### Input variables

Input sources are openly available online and are summarized in the following Section and in Table 1.

**Table 1:** Summary of data sources and descriptions of the Metair dataset.

Dimension	Source	Data description	Spatial coverage
Classified air quality station metadata	New South Wales Government^20^	Metadata on air quality stations in Australia	Australia
	European Environment Agency (EEA)^17^	Metadata on air quality stations in Europe	Europe
	Environment and Climate Change Canada (ECCC)^22^	Metadata on air quality stations in Canada	Canada
	Environmental observatory of the National Institute for Environmental Studies^24^	Metadata on air quality stations in Japan	Japan
	Environment Canterbury Regional Council^27^	Metadata on air quality stations in New Zealand	New Zealand
	Department of Forestry, Fisheries and the Environment Republic of South Africa^28^	Metadata on air quality stations in South Africa	South Africa
	Environmental Protection Agency (EPA)^29^	Metadata on air quality stations in the United States	United States
Unclassified air quality station metadata	Instituto de Energia e Meio Ambiente (IEMA)^21^	Metadata on air quality stations in Brazil	Brazil
	China National Environmental Monitoring Centre (CNEMC)^23^	Metadata on air quality stations in China	China
	Sistema Nacional de información de la calidad del aire (SINAICA); Secretaría de Medio Ambiente e Recursos Naturales^25^	Metadata on air quality stations in Mexico	Mexico
	Sistema de Monitoreo Atmosférico de la Ciudad de México (SIMAT); Secretaría del Medio Ambiente del Gobierno de la CDMX^26^	Metadata on air quality stations in Mexico City region	Mexico City region
	OpenAQ^30^	Locations of governmental air quality stations monitoring PM for countries from Africa, Asia, America, and Oceania	Global
Land cover	European Space Agency (ESA)^31^	Land cover data from the ESA WorldCover 10 m 2021 v200 product	Global
Industrial plants	Global Energy Observatory^32^	Data on industrial plants from the Global Power Plant Database	Global
	Global Energy Monitor^33–37,39^	Data from the Global Coal Plant Tracker, the Global Coal Mine Tracker, the Global Oil and Gas Plant Tracker, the Global Steel Plant Tracker, the Global Bioenergy Power Tracker, and the Global Database of Cement Production Assets and Upstream Suppliers	Global
	EEA^38^	European Pollutant Release and Transfer Register	Europe
Industrial emissions	Copernicus Atmosphere Monitoring Service (CAMS)^43,44^	2022 CAMS-GLOB-ANT NO_*x*_, SO_2_, NMVOC, and NH_3_ yearly emissions from selected sectors	Global
Air pollution	Global High-Resolution Air Pollution (GHAP) datasets^41,42^	1-km estimates of PM_2.5_ and CO	Global
Population density	Center for International Earth Science Information Network, Columbia University^45^	2020 1-km population density (Revision 11), Gridded Population of the World dataset, v 4 (GPWv4)	Global
Other satellite data	SatCLIP^46^	Various spatial patterns	Global

##### Air quality station labels

**Fig. 1:**
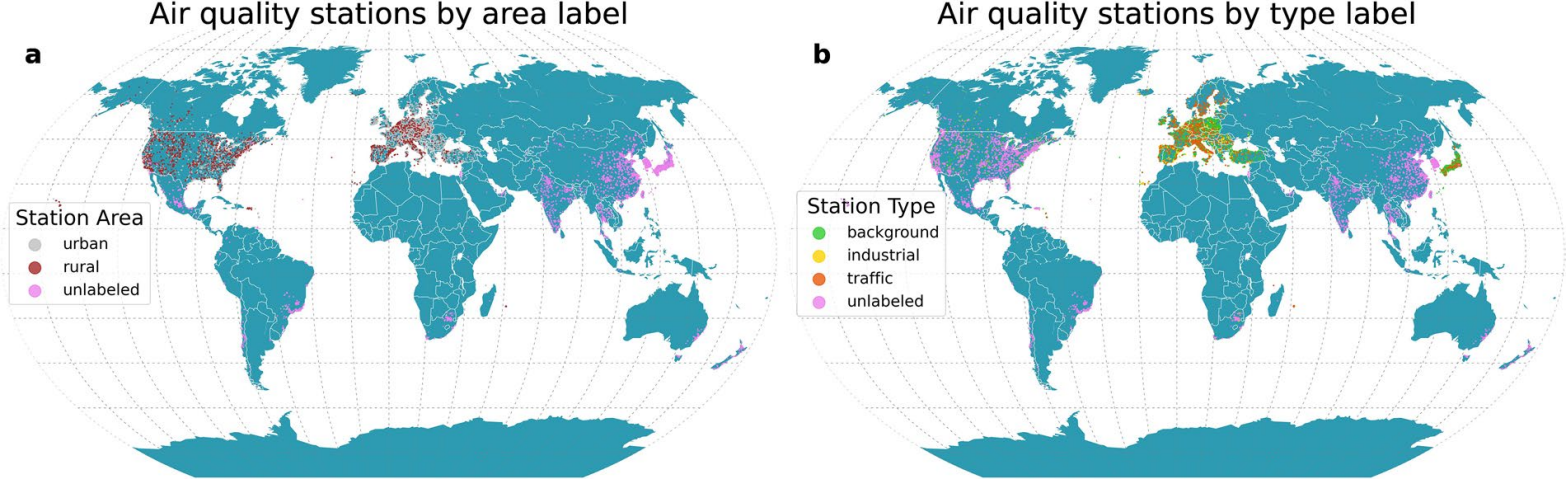
Location of (**a**) classified (urban, rural) and unlabeled air quality stations based on air quality station area characteristics and (**b**) classified (background, industrial, traffic) and unlabeled air quality stations based on air quality station type.

The metadata of air quality stations are derived from different international sources^17,20–29^. These metadata are used to train the model on the target classification outputs (Fig. 1). Numerous countries do not have an integrated air quality network, particularly in the Global South. We retrieve locations of governmental air quality stations monitoring PM for other countries from OpenAQ (version 2)^30^. As some air quality stations may have multiple monitors or samplings for a pollutant, we retrieve unique locations of air quality stations. To increase the number of unique locations, we relax the restriction on the considered pollutants. As the air quality station characteristics would be the same, for some countries we consider governmental locations monitoring fine particulate matter (PM_2.5_), coarse particulate matter (PM_10_), and total particulate matter mass.

##### Land cover

**Fig. 2:**
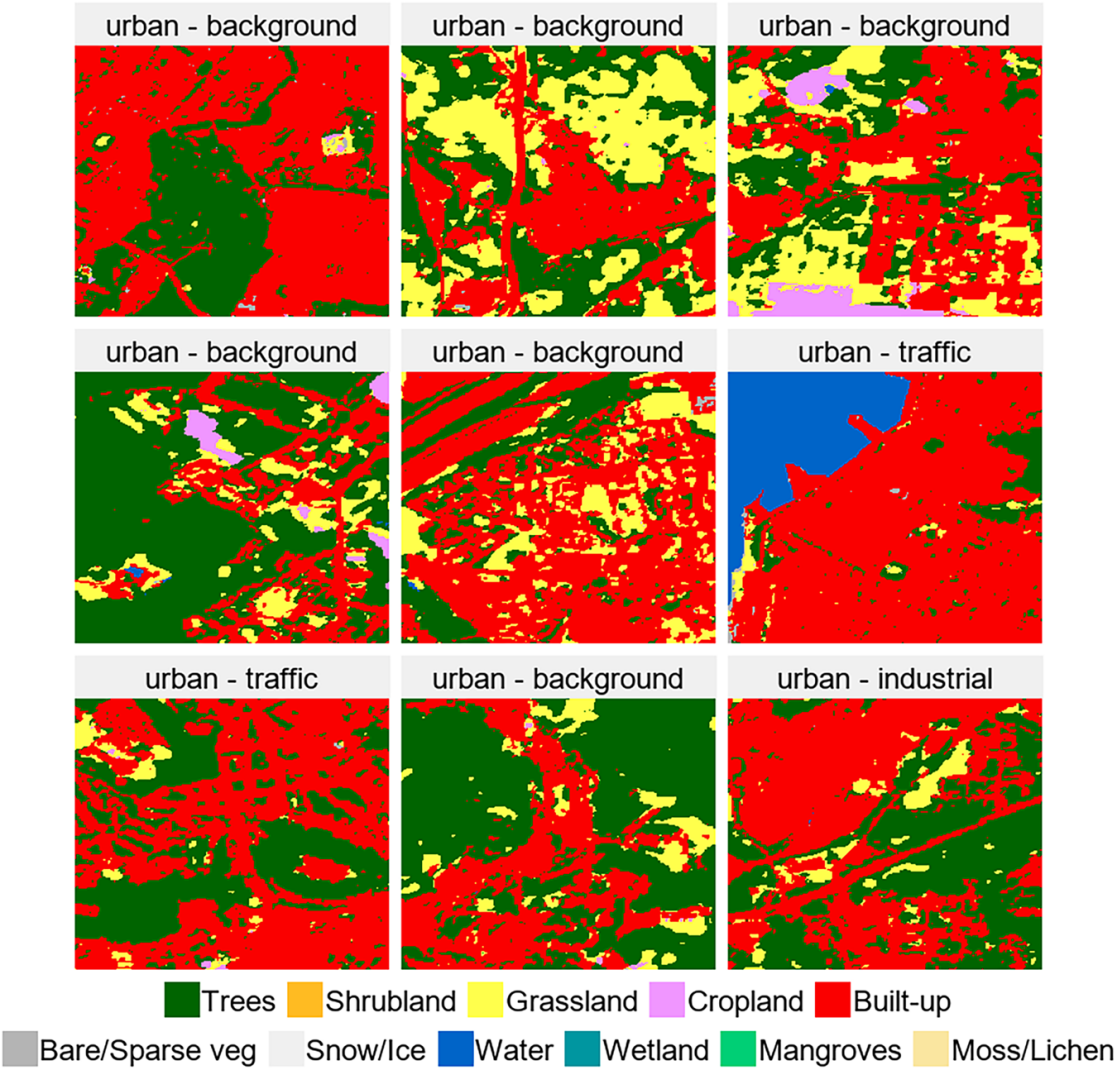
Land cover input samples. ESA WorldCover 10m resolution land cover data at 9 classified air quality stations’ locations, with overlaying corresponding station area and type labels.

Land cover data come from the European Space Agency (ESA) WorldCover 10 m 2021 v200 product (0.0000834^°^ x 0.0000834^°^)^31^. As an example, Fig. 2 shows a sample of the land cover images with corresponding air quality station area characteristics and type labels. Land cover data are useful for identifying features surrounding monitoring stations that are crucial for classification, such as major roads, large industrial facilities, cropland, green spaces, or residential neighborhoods.

##### Industrial sources

**Fig. 3:**
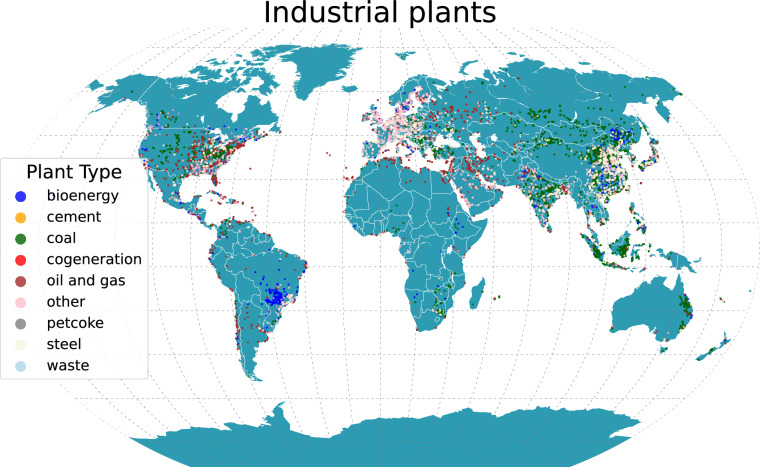
Locations of industrial plants affecting PM by plant type.

Data on large operating industrial plants by type come from several sources, such as the Global Power Plant Database v 1.3.0 (2021-06-02 release)^32^ by the World Resources Institute and others, the Global Coal Plant Tracker (July 2023 release)^33^, the Global Coal Mine Tracker (October 2023 release)^34^, the Global Oil and Gas Plant Tracker (August 2023 release)^35^, the Global Steel Plant Tracker (March 2023 release)^36^, the Global Bioenergy Power Tracker V1 (November 15, 2023 release)^37^ by the Global Energy Monitor, the EEA European Pollutant Release and Transfer Register v 9.0 (May 2023 release)^38^, and the Global Database of Cement Production Assets and Upstream Suppliers (October 2023 release)^39,40^. To focus on industrial plants affecting PM, we harmonize plant type information for 31,000 plants into the following 9 categories, as shown in Fig. 3: bioenergy, cement, coal, cogeneration, oil and gas, other, petcoke, steel, and waste. We flag stations located within 20 km of an industrial plant affecting PM, keeping track of its source type. The inclusion of industrial plant data is intended to help the model identify industrial stations and distinguish them from other classes.

##### Air pollution

Proxies of air pollution are built based on the 1-km Global High-Resolution Air Pollution (GHAP) v 1 estimates of PM_2.5_^41^ and carbon monoxide (CO) annual concentrations^42^. We apply bilinear interpolation to get an interpolated value of 2017-2022 PM_2.5_ (excluding 2020) at the air quality stations’ locations. We attribute the 2022 1-km annual global CO estimate cell value in which the station location falls. This spatial attribution serves two key purposes. First, it provides an external, harmonized proxy of pollution intensity that is independent of national monitoring practices. Second, combining these estimates with observed annual concentration levels at the stations allows us to characterize typical pollution patterns — such as high levels of CO, typical of traffic sources, or low levels of PM indicative of background sources.

We also obtain 2022 CAMS Global Anthropogenic (CAMS-GLOB-ANT) v 6.2 yearly, gridded emissions for black carbon (BC), nitrogen oxides (NO_*x*_), sulfur dioxide (SO_2_), non-methane volatile organic compounds (NMVOC), and ammonia (NH_3_)^43,44^ through the Copernicus Atmosphere Monitoring Service (CAMS) portal. The data have a monthly temporal resolution and a spatial resolution of 0.1^°^, and are expressed in Teragrams. We attribute the 10-km annual sectoral emission values in which the station location falls.

##### Population

Population data are taken from the 2020 1-km population density (Revision 11) product from the Gridded Population of the World dataset, v 4 (GPWv4), developed by the Center for International Earth Science Information Network, Columbia University^45^. We attribute to air quality stations the annual population density cell value in which the station location falls. Population is a relevant predictor of station classification, as it helps distinguish between urban and rural contexts. Densely populated areas are more likely to host traffic-related stations, while sparsely populated regions are typically associated with rural background monitoring. It provides the classifier with an additional layer of context supporting the learning of spatial patterns linked to human activity and emission sources.

##### Other satellite data

We proxy geographical information at our locations of interest through the Satellite Contrastive Location-Image Pretraining (SatCLIP) encoder^46^. This embedding captures spatial patterns such as urban structure, vegetation cover, infrastructure density, and land use type. Including SatCLIP-derived features allows the model to incorporate rich visual information that may not be explicitly captured in traditional metadata, improving classification performance, especially in ambiguous or mixed-use settings.

### Data processing pipeline

#### Data harmonization

Air quality stations’ metadata derived from diverse sources are harmonized into a unique dataset based on the EEA labeling systems. See Tables 2 and 3 for more details on original labels by country and network. Our final dataset consists of 14970 unique locations. Regarding the station type metadata, the dataset portion of classified air quality stations includes 6777 labeled stations, i.e., 4440 background, 1525 traffic, and 852 industrial stations. Regarding the area characteristics metadata, 2026 air quality stations are classified as rural, and 6919 air quality stations as urban. While, we collect 8193 and 6025 unlabeled locations regarding the two categories, respectively. See Table 4 for more insights. Therefore, the dataset is unbalanced towards background and urban locations. We associate a land cover image to each air quality station location. We crop satellite images into a square region centered on the station location, using a 0.01^°^-buffer as side length, i.e., roughly 2000 m  × 2000 m (Fig. 2).

**Table 2:** Harmonization of classified air quality stations: type.

Country / Network	Raw variable name	Type labels
CAN	Site type	general population exposure (PE); regional backgrounds (RB); transportation source-influenced (T); point source-influenced
CAN	Land use	residential (R); commercial (C); industrial (I); parks (P); water (W); agriculture (A); forested (F); open (O)
EEA	Air quality station type	background; industrial; traffic
EPA	Land use	industrial
EPA	Monitoring objective	general/background; population exposure; welfare-related impacts; source-oriented
EPA	Networks	road-side
NZL	Site type	residential; NES site; traffic; industrial; coastal
JAP	Station classification	background (1); traffic (2); other (9)
ZAF	Station target	residential - low income; residential - medium/upper income; industrial; traffic - roadside; urban; background; peri-urban; domestic & industrial; traffic - street canyon; industrial & residential

Note: raw variable names have been modified to improve clarity. E.g., from STA_Type to Station type. Labels have been changed to lowercase.

**Table 3:** Harmonization of classified air quality stations: area characteristics.

Country / Network	Raw variable name	Area characteristics labels
CAN	Urbanization	large urban area (LU); medium urban area (MU); small urban area (SU); non-urban (rural) area (NU)
EEA	Air quality station area	suburban; rural; urban; rural-regional; rural-nearcity; rural-remote
EPA	Local setting	urban and center city; suburban; rural
ZAF	Station target	residential - low income; residential - medium/upper income; industrial; traffic - roadside; urban; background; peri-urban; domestic & industrial; traffic - street canyon; industrial & residential

Note: labels have been changed to lowercase.

**Table 4:** Summary of classified air quality station metadata by air quality station type and area characteristics.

Area	Type				
	Background	Industrial	Traffic	Unlabeled	Totals
**Urban**	2542	596	1269	2512	6919
**Rural**	1034	228	15	749	2026
**Unlabeled**	824	28	241	4932	6025
**Totals**	4400	852	1525	8193	14970

In addition to land cover, for each air quality station, we gather additional metadata that are fed into the model to further inform the prediction.

### Classification model architecture

Our methodology employs a two-stage hierarchical classification approach motivated by the inherent structure of air quality station classification. Station area characteristics (urban vs. rural) represent fundamental environmental contexts that influence pollution source identification. Therefore, we first classify stations by area characteristics, then exploit these predictions as additional features for source type classification (background vs. non-background), which is a comparably harder task to solve.

This modeling decomposition addresses two key challenges: first, the class imbalance problem, where background stations significantly outnumber non-background stations; and second, the conditional dependency between area and source characteristics — rural stations are predominantly background (see Table 4) while urban stations exhibit a wider range of source types.

### Multi-modal fusion architecture

**Fig. 4:**
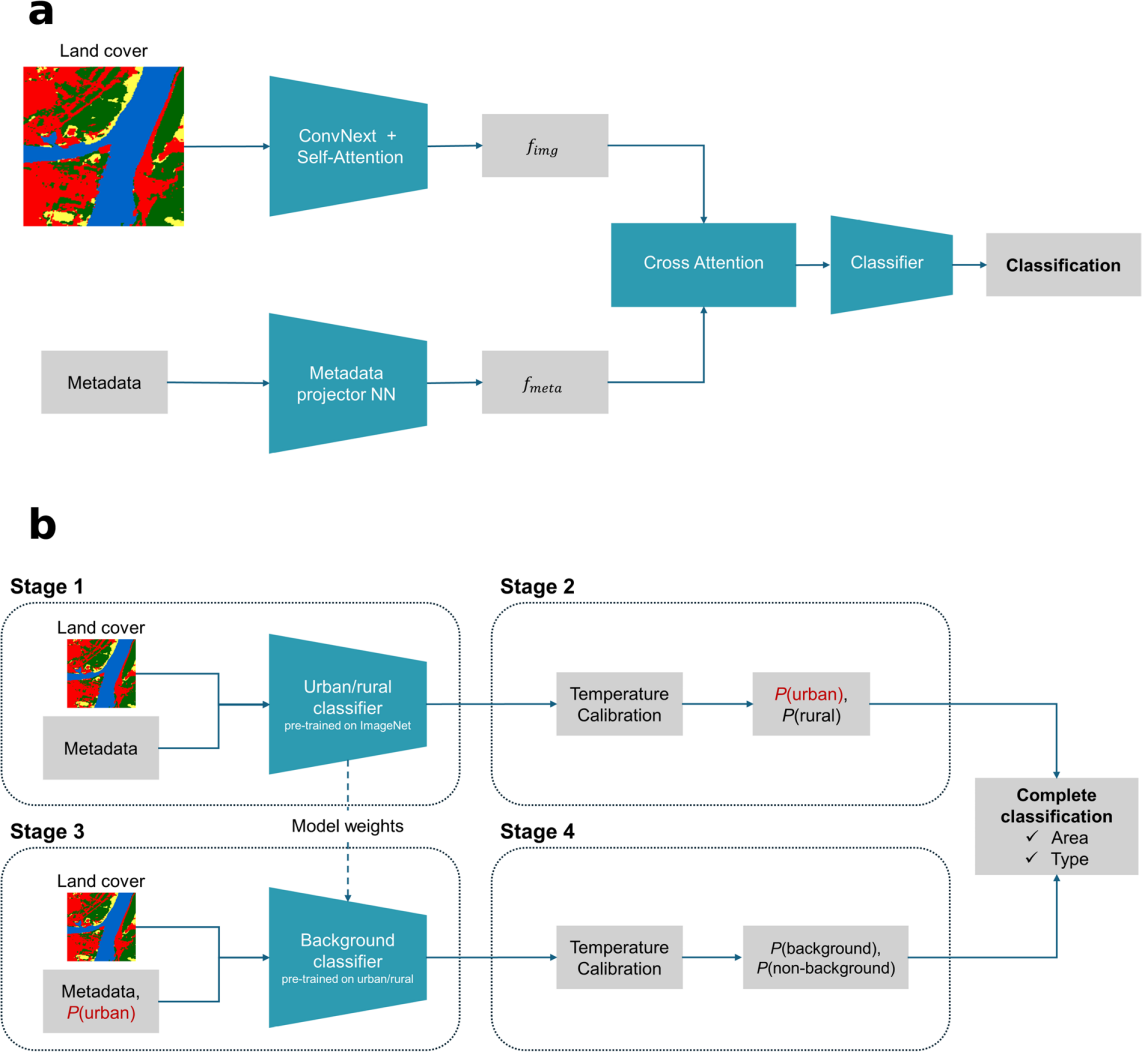
Diagram of (**a**) modeling pipeline and (**b**) detail of the modeling stages.

Accurately classifying air quality stations requires integrating two fundamentally different types of information: visual patterns from land cover imagery and quantitative environmental indicators. Traditional approaches would simply concatenate these features, but this fails to capture the complex relationships between visual context and environmental measurements. For instance, the relevance of industrial distance measurements depends heavily on the surrounding land use patterns visible in satellite imagery. To address this challenge, we developed a cross-attention^47^ fusion architecture that allows the model to dynamically weight different information sources based on their relevance to each specific location. The architecture is illustrated Fig. 4 panel (a), and consists of three main components:

#### Visual Feature Extraction

Inspired by recent work on computer vision^48^, we employ a ConvNext-*small*^49^ architecture pre-trained on the ImageNet^50^ dataset as our backbone, enhanced with linear self-attention layers^51^ to capture long-range spatial dependencies. This processes 224 × 224 land cover images to extract a 64-dimensional feature vector encoding spatial patterns of land cover, such as urban infrastructure, vegetation coverage, and industrial areas.

#### Metadata Processing

Environmental, geo-spatial, and demographic indicators provide crucial quantitative context that may not be visually apparent. We leverage a multidimensional metadata feature vector containing:

- distance to nearest industrial facilities (log-transformed and normalized);
- PM_2.5_ and CO concentration estimates from satellite-derived datasets;
- population density from gridded demographic data;
- emissions within the station’s grid cell;
- SatCLIP^46^ location embeddings capturing broader geographical context.

These 284-dimensional metadata features are projected to the same 64-dimensional feature space as the visual features through a neural network with SiLU^52,53^ activations and layer normalization^54^, ensuring compatible representations for feature fusion.

#### Cross-Attention Fusion

Rather than simple feature concatenation, our cross-attention mechanism enables the model to selectively focus on relevant metadata based on the visual context. For example, when the image shows dense urban development, the model may learn to emphasize population density and traffic-related emissions, while for rural areas it may prioritize distance to industrial sources. This adaptive fusion strategy allows the model to make context-aware decisions that improve classification accuracy across diverse geographical regions and station types.

The cross-attended features are then processed by a final classification head to produce the station type predictions. This architecture’s flexibility is designed to handle the heterogeneous nature of global air quality monitoring networks, where the same visual patterns may indicate different station types depending on local environmental conditions.

### Training methodology and transfer learning

Our two-stage training approach, illustrated in Fig. 4 panel (b), is designed to leverage the hierarchical nature of air quality station classification while addressing data availability constraints.

#### Stage 1: (Urban/Rural Classification)

The model is trained on approximately 5700 labeled stations using cross-entropy loss with label smoothing, to address dataset imbalance and avoid overconfident estimations. We employ extensive regularization (dropout, weight decay) and data augmentation including geometric transformations, and photometric distortions to improve generalization across diverse geographical regions and imagery conditions. In this stage, the model is fine-tuned from a model pre-trained on Imagenet classification.

#### Stage 2 (Background/Non-background classification)

The architecture processes identical visual inputs supplemented with urban/rural probability estimates from Stage 1. This design reflects the conditional relationship between area characteristics and source types, providing the model with hierarchical context for improved discrimination. Transfer learning from Stage 1 initializes the backbone and attention layers, leveraging learned spatial feature representations while adapting the classification head for the new task. This approach addresses the limited availability of background/non-background labels compared to urban/rural annotations. For this stage, we fine-tune the model trained on Stage 1, leveraging the specialized features it learned for understanding the air quality station classification domain.

#### Temperature calibration

Both stages employ post-training temperature calibration^55^ to improve probability reliability and reduce overconfidence. A learnable temperature parameter *T* scales the logits before softmax normalization, optimized on validation data to minimize negative log-likelihood. This ensures that predicted probabilities accurately reflect classification confidence, critical for uncertainty-aware applications in environmental monitoring. The complete training pipeline code is included in a Zenodo repository^56^.

## Data Records

This article presents a global dataset of original and estimated air quality stations metadata, called Metair. The final dataset is available online in a Zenodo repository^56^. This repository contains 3 folders and a file, as follows:

- dataset.zip: a compressed folder with the Metair global dataset of air quality stations’ harmonized metadata and supplementary files.
- model_input.zip: a compressed folder with land cover images and additional metadata as model input;
- code.zip: a compressed folder with the scripts for replication;
- README.md: a text file describing code and input sources.

**Fig. 5:**
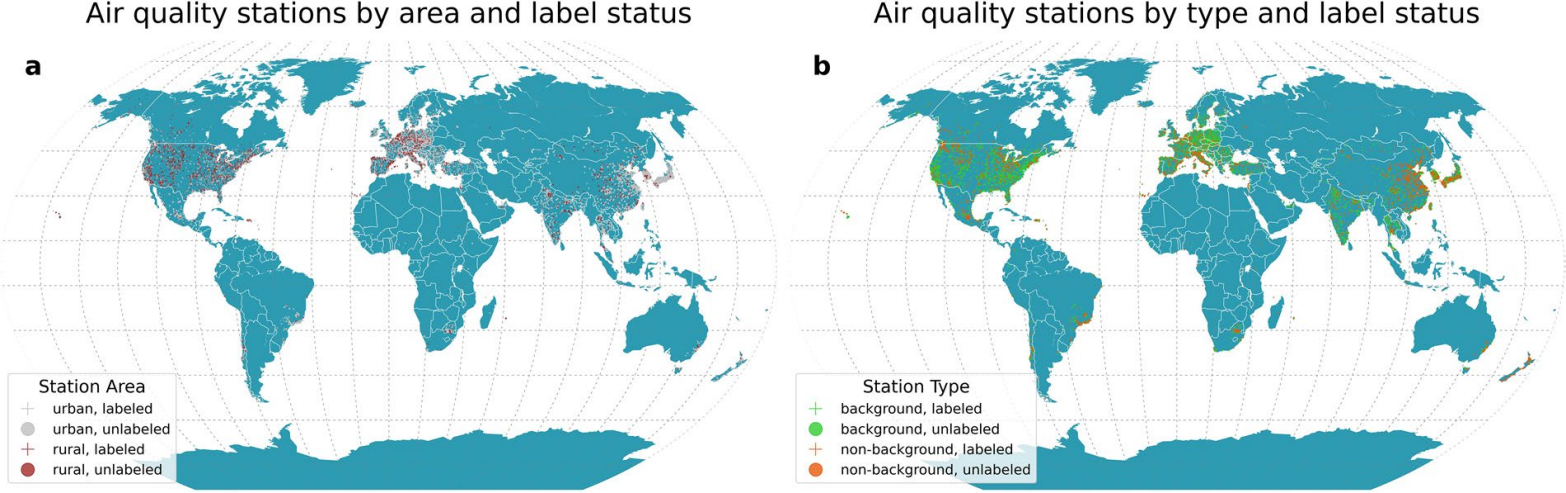
Location of classified and estimated air quality stations metadata by (**a**) area characteristics (urban, rural) and (**b**) type (background, non-background).

Apart from the README file, we provide all data in a comma-separated (CSV) format, readable by several widely used open-source and commercial software programs. They can also be read into programming language environments, e.g., Python and R, through suitable functions. The main file of interest, dataset_v_1.csv, is contained in dataset.zip. It comprises the following variables, as specified in Table 5. For each station, an identifier identifies the air quality station (unique_id), a categorical variable names the pollutant measured at such station (pollutant), a three-letter country ISO code (iso) locates it nationally, while its World Geodetic System 1984 (WGS84) geographic coordinates in degrees (longitude, latitude) and elevation in meters (elevation) provide its position. Stations are further classified by the air quality station area characteristics (urban, rural) and type (background, non-background) to capture the local pollution context. Two binary flags indicate whether the type and area characteristics classifications have been directly attributed by an institutional agency such as a governmental air quality network (labeled_type, labeled_area = 1) or estimated by our model (= 0), ensuring transparency about the origin of the data. Note that official labels, available in the metadata_[iso]_v_1.csv files, might have been modified, as explained in the Methods Section. Together, these variables allow analysis of spatial and contextual factors influencing air quality measurements. A spatial representation of the final harmonized dataset is provided in Fig. 5.

**Table 5:** Variables of the Metair dataset.

Variable name	Description	Unit
unique_id	Air quality station location alpha-numeric identifier	Categorical
pollutant	Pollutant name	Categorical
iso	3-letter country isocode	Categorical
longitude	Air quality station longitude	Degrees
latitude	Air quality station latitude	Degrees
elevation	Air quality station elevation	Meters
area	Air quality station area. Values of area are: urban, rural	Categorical
type	Air quality station type. Values of type are: background, non-background	Categorical
labeled_area	Indicating if the air quality station area characteristic is provided by authorities (1) or estimated (0)	Dummy
labeled_type	Indicating if the air quality station type is provided by authorities (1) or estimated (0)	Dummy

Table 6 provides a summary of the labeled and predicted air quality station metadata by area characteristics and station type.

**Table 6:** Summary of labeled (L) and predicted (P) and total (T) air quality station metadata by air quality station type and area characteristics.

Area		Type						
		Background		Non-background		Totals		
		L	P	L	P	L	P	T
**Urban**	**L**	2542	1999	1865	513	4407	2512	6919
	**P**	811	3257	263	1382	1074	4639	5713
**Rural**	**L**	1034	630	243	119	1277	749	2026
	**P**	13	252	6	41	19	293	312
**Totals**	**L**	3576	2629	2108	632	5684	3261	8945
	**P**	824	3509	269	1423	1093	4932	6025
	**T**	4400	6138	2377	2055	6777	8193	14970

The dataset.zip folder also contains modeling_raw.zip: a compressed folder with the raw metadata files together with the modeling variables (metadata_[iso]_v_1.csv).

Modeling variables are provided in a separate unique file, dataset_modeling_v_1.csv, are described in Table 7, and consist in what follows. bilinear_pm25_20[XX] and cell_pm25_20[XX] represent annual 1-km PM_2.5_ concentrations (*μg*/*m*^3^) for year 20[XX] from 2017 to 2022, estimated using bilinear interpolation and direct grid cell values, respectively (both numeric). The variables bilinear_pm25_mean_2017_2022 and cell_pm25_mean_2017_2022 contain the corresponding multi-year average PM_2.5_ concentrations over 2017-2022 (numeric). Additional variables include cell_pop_density_2020 for population density per grid cell in 2020 (persons/km^2^, numeric), cell_[poll]_emi_[sector]_2022 for grid-cell pollutant-specific sectoral emissions in 2022 (BC, NO_*x*_, SO_2_, NMVOC, NH_3_, numeric), and cell_co_2022 for 1-km CO concentrations at the grid cell in 2022 (numeric). Station site classification variables comprise the model-derived probabilities of being urban/rural in urban_probability and rural_probability, respectively, along with their numeric equivalents full_urban_prob and full_rural_prob (0-1 range). The binary prediction of the area classification is given by full_predicted_area (0 = urban, 1 = rural, dummy) with corresponding categorical labels in full_predicted_area_label (urban, rural). Regarding the station type classification, full_background_prob and full_nonbackground_prob express the probability that a site is background/non-background (0-1, numeric), while full_predicted_type is the binary prediction (0 = background, 1 = non-background), and full_predicted_type_label is the categorical label for the predicted type (background, non-background). Model performance metrics include full_urban_rural_accuracy and full_background_accuracy, representing classification accuracy scores for urban/rural and background/non-background predictions, respectively (both numeric). For transparency, they are also provided together with original metadata files, under the metadata_modeling_raw.zip folder: non-original variables are flagged with the _metair suffix.

**Table 7:** Additional variables of the Metair dataset.

Variable name	Description	Unit
bilinear_pm25_20[XX]	1-km PM_2.5_ concentration for year 20[XX], estimated with bilinear interpolation	Numeric
cell_pm25_20[XX]	1-km PM_2.5_ concentration for year 20[XX] from the grid cell	Numeric
bilinear_pm25_mean_2017_2022	Multi-year average 1-km PM_2.5_ concentration with bilinear interpolation from 2017 to 2022	Numeric
cell_pm25_mean_2017_2022	Multi-year average 1-km PM_2.5_ concentration (cell value from 2017 to 2022)	Numeric
cell_pop_density_2020	2020 population density in the grid cell	Numeric
cell_[poll]_emi_[sector]_2022	2022 sectoral emissions from the grid cell	Numeric
cell_co_2022	2022 1-km CO concentration from the grid cell	Numeric
urban_probability	Model-derived probability of being urban	Numeric
rural_probability	Model-derived probability of being rural	Numeric
full_urban_prob	Probability of being urban (0-1)	Numeric
full_rural_prob	Probability of being rural (0-1)	Numeric
full_predicted_area	Binary prediction (0 = urban, 1 = rural)	Dummy
full_predicted_area_label	Label for area prediction (urban, rural)	Categorical
full_background_prob	Probability that the site is background	Numeric
full_nonbackground_prob	Probability that the site is non-background	Numeric
full_predicted_type	Binary prediction (0 = background, 1 = non-background)	Dummy
full_predicted_type_label	Label for the predicted type (background, non-background)	Categorical
full_urban_rural_accuracy	Accuracy score for urban/rural classification	Numeric
full_background_accuracy	Accuracy score for background/non-background classification	Numeric

model_input.zip contains labeled.zip and unlabeled.zip, compressed folders with land cover portions for labeled and unlabeled air quality stations’ location, plus metadata_final.csv, a CSV file to be fed into the model.

**Fig. 6:**
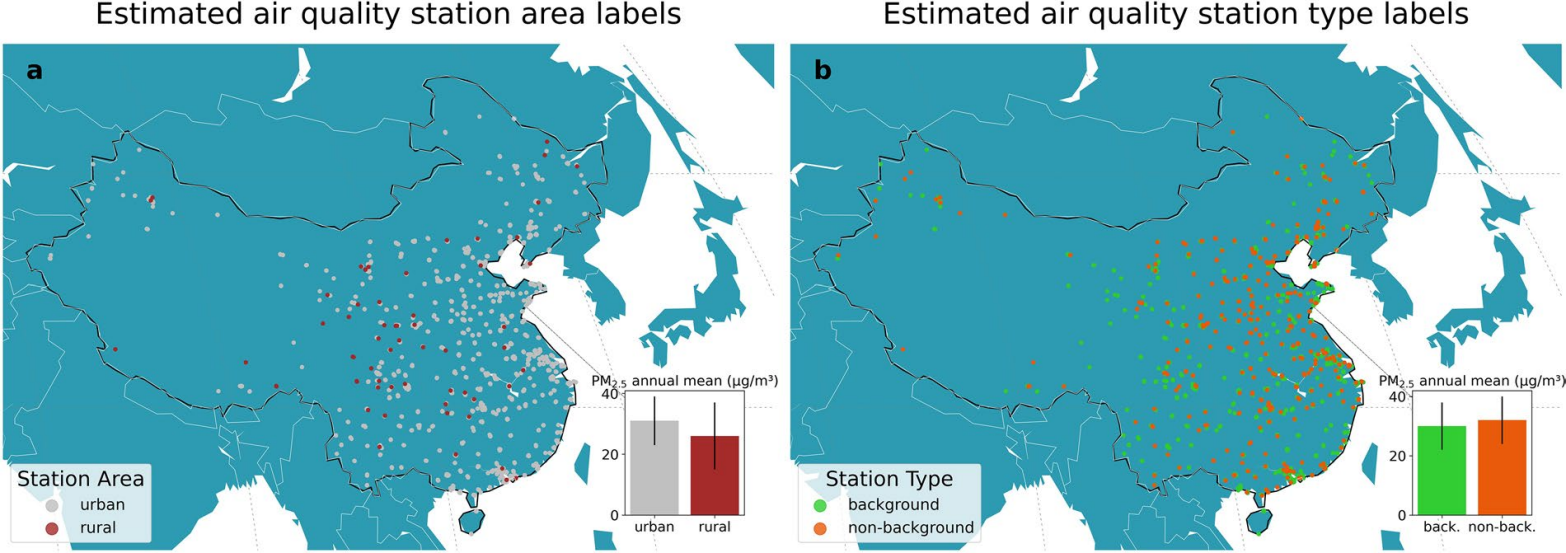
Location of estimated PM_2.5_ air quality stations metadata by (**a**) area characteristics (urban, rural) and (**b**) type (background, non-background, also shortened as back. and non-back.) for mainland China. Side barplots show 2024 PM_2.5_ annual mean and standard deviation in *μg*/*m*^3^ by label based on measurements^57,58^ provided by the cited authors.

In Fig. 6, we focus on mainland China air quality stations’ locations by area characteristics and type, the most populous country with unclassified stations. In addition, we show 2024 annual averages by predicted station labels^57,58^. As expected, rural locations display a lower annual average compared to urban areas (26.3 vs 31.1 micrograms per cubic meter, *μg*/*m*^3^), as well as background against non-background locations (30.1 vs 32.6 *μg*/*m*^3^).

### Possible applications

Environmental agencies strategically place new monitoring stations based on emission sources and coverage needs, among the others. Our primary objective is to harmonize the classification of existing air quality stations globally. Beyond that, the proposed model can serve as a decision-support tool for planning new monitoring sites. When budget constraints limit the number of stations that can be deployed for a specific purpose, planners can use the model to evaluate if a prospective location’s predicted classification aligns with the intended monitoring objective. For instance, if a site planned for background monitoring is predicted as “urban non-background”, this mismatch signals potential interference from local sources and suggests reconsidering the placement. By identifying such inconsistencies before deployment, agencies can optimize station networks and avoid costly misplacements. This functionality is available with the current version of the model.

### Known limitations

Given the fragmented data management of air quality stations and limited accessibility of their data globally, some locations may not be included in this dataset or may display imprecise geographical position due to inaccuracies at the source. These shortcomings will be addressed in future public releases as more locations and metadata are recovered.

Our classification model is designed specifically for existing monitoring stations rather than arbitrary point locations. With sparse air quality networks worldwide, extending the model to predict classifications at any geographic coordinate would be computationally expensive and unnecessary given the limited number of actual stations. However, a feasible future extension could focus on densely populated and urban areas, where the higher concentration of monitoring needs would justify the computational cost. Such an extension could support emerging applications including low-cost sensor networks and citizen science campaigns, though these remain outside our current scope.

## Technical Validation

### Dataset composition and experimental setup

Our validation methodology employs stratified train-validation splits to ensure representative sampling across station types and geographical regions. For urban/rural classification, we utilize 8298 training samples and 921 validation samples (90%/10% split). The training data exhibits class imbalance with 76.5% urban stations (6345 samples) and 23.5% rural stations (1953 samples), reflecting the real-world distribution of air quality monitoring infrastructure. For background/non-background classification, we train on 6849 stations comprising 4563 background stations (66.7%) and 2286 non-background stations (33.3%, including 1366 traffic and 920 industrial stations). The validation set contains 759 stations with a similar distribution (506 background, 253 non-background).

### Classification performance

Model performance is evaluated using accuracy and F1 score metrics. The F1 score represents the harmonic mean of precision and recall ($F1=2\times \frac{\,{\rm{precision}}\times {\rm{recall}}}{{\rm{precision}}+{\rm{recall}}}$), providing a balanced measure that accounts for both false positives and false negatives, particularly important for unbalanced datasets as ours.

#### Urban/Rural classification

The model achieves an F1 score of 0.931 on validation data. This demonstrates strong generalization despite the significant class imbalance, highlighting the relative ease of learning this binary classification.

#### Background/Non-background classification

This more challenging task achieves 0.774 F1 score on validation data, reflecting the inherent difficulty of distinguishing background from source-influenced stations using land cover imagery and environmental metadata. The hierarchical approach, incorporating urban/rural probabilities from Stage 1, provides contextual information that improves source type discrimination compared to single-stage classification approaches.

### Temperature calibration and uncertainty quantification

Post-training calibration significantly improves probability reliability for both classification stages. The urban/rural classifier requires minimal calibration (temperature = 1.03), indicating well-calibrated initial predictions. In contrast, the background classifier benefits from substantial calibration (temperature = 2.11), reflecting the increased difficulty of source type discrimination and the model’s initial overconfidence. Temperature calibration optimizes the cross-entropy loss on validation data, ensuring that predicted probabilities accurately reflect classification confidence. This calibration is crucial for uncertainty-aware applications in environmental monitoring, where prediction confidence directly informs data quality assessments.

## Data Availability

The METAIR dataset created in this study is openly available in a Zenodo repository at 10.5281/zenodo.15680868. Secondary data used as input are openly available online or upon request. The air quality station metadata are available at https://www.environment.nsw.gov.au/topics/air/monitoring-air-quality for Australia, at https://energiaeambiente.org.br/qualidadedoar/ for Brazil, at https://datadonnees.ec.gc.ca/data/air/monitor/national-air-pollution-surveillance-naps-program/ for Canada, at http://www.cnemc.cn for China, at https://www.eea.europa.eu/ for Europe, at https://tenbou.nies.go.jp/ for Japan, at https://sinaica.inecc.gob.mx/ for Mexico, at http://www.aire.cdmx.gob.mx/ for Mexico City, at https://www.lawa.org.nz/download-data##air for New Zealand, at https://saaqis.environment.gov.za/ for South Africa, at https://aqs.epa.gov/ for the United States, and at https://openaq.org/ (version 2) for remaining countries. The ESA WorldCover 10 m 2021 v200 land cover data are available at 10.5281/zenodo.7254221. The high-resolution estimates of PM_2.5_ and CO from the Global High-Resolution Air Pollution (GHAP) v 1 datasets are available at 10.5281/zenodo.10800980 and 10.5281/zenodo.14207363, respectively. High-resolution population density from the Gridded population of the world, v 4 (GPWv4): Population density, revision 11 is available at 10.7927/H49C6VHW. CAMS-GLOB-ANT v 6.2 global sectoral emission data are available at https://eccad.aeris-data.fr/. Industrial plants’ locations by the Global Energy Observatory and the Global Energy Monitor are available at http://datasets.wri.org/dataset/globalpowerplantdatabase and https://globalenergymonitor.org/, respectively. The Global Database of Cement Production Assets and Upstream Suppliers is available at 10.5061/dryad.6t1g1jx4f. The European Pollutant Release and Transfer Register v 9.0 is available at https://sdi.eea.europa.eu/catalogue/srv/api/records/9405f714-8015-4b5b-a63c-280b82861b3d.

## Code availability

The code is available online in a Zenodo repository at 10.5281/zenodo.15680868 under the code.zip compressed folder.
